# P-625. Missed opportunities for vaccination among healthcare-seeking children–Kyrgyzstan, 2023

**DOI:** 10.1093/ofid/ofae631.823

**Published:** 2025-01-29

**Authors:** Gulzada Dadanova, Aisuluu Kubatova, Roberta Horth, Dilyara Nabirova, Gulbara Ishenapisova, Dinara Otorbaeva

**Affiliations:** Central Asia Advanced Field Epidemiology Training Program, Bishkek, Bishkek, Kyrgyzstan; Ministry of Health of the Kyrgyz Republic, National Institute of Public Health, Bishkek, Kyrgyzstan, Bishkek, Bishkek, Kyrgyzstan; US Centers for Disease Control and Prevention, Dulles, Virginia; CDC Central Asia office, Almaty, Almaty, Kazakhstan; Republican Center of immunoprophylaxis Kyrgyz Republic, Bishkek, Bishkek, Kyrgyzstan; Department of Disease Prevention and State Sanitary and Epidemiological Supervision, Bishkek, Kyrgyzstan, Bishkek, Bishkek, Kyrgyzstan

## Abstract

**Background:**

High vaccination coverage is critical to prevent disease transmission. In Bishkek, Kyrgyzstan (population: 1.15 million), coverage for many childhood vaccines had dropped to < 90% in 2022. Opportunities for vaccination were being missed at child healthcare visits. Understanding the burden and reasons for these missed opportunities was needed to increase coverage.

**Figure 1.**
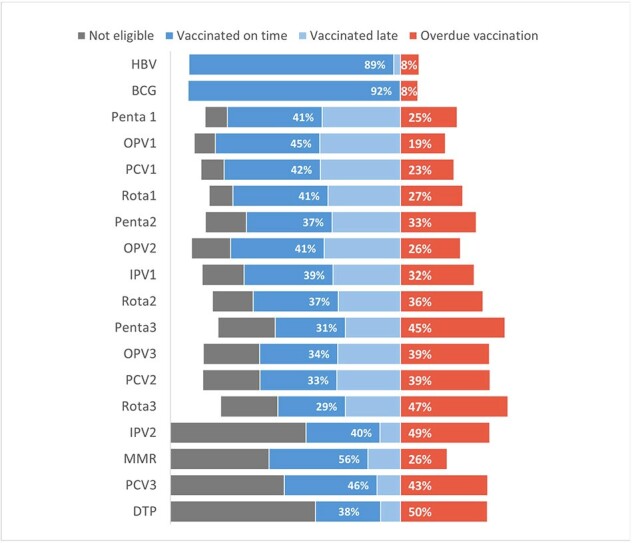
Missed opportunities for vaccination by vaccine type among healthcare-seeking children in Kyrgzystan, 2023

**Methods:**

We conducted a cross-sectional study in Bishkek in 2023 using the Missed Opportunities for Vaccination (MOV) World Health Organization protocol. We selected 33 primary care clinics and interviewed 20 caregivers of < 30 months olds in each. We abstracted vaccine history from medical records. MOV was calculated as the number of children not receiving a vaccine among those eligible for vaccination (defined as missing a dose at the start of the visit and no vaccine contraindications). The p-values from Poisson regression Wald tests are shown.

**Table 1.**
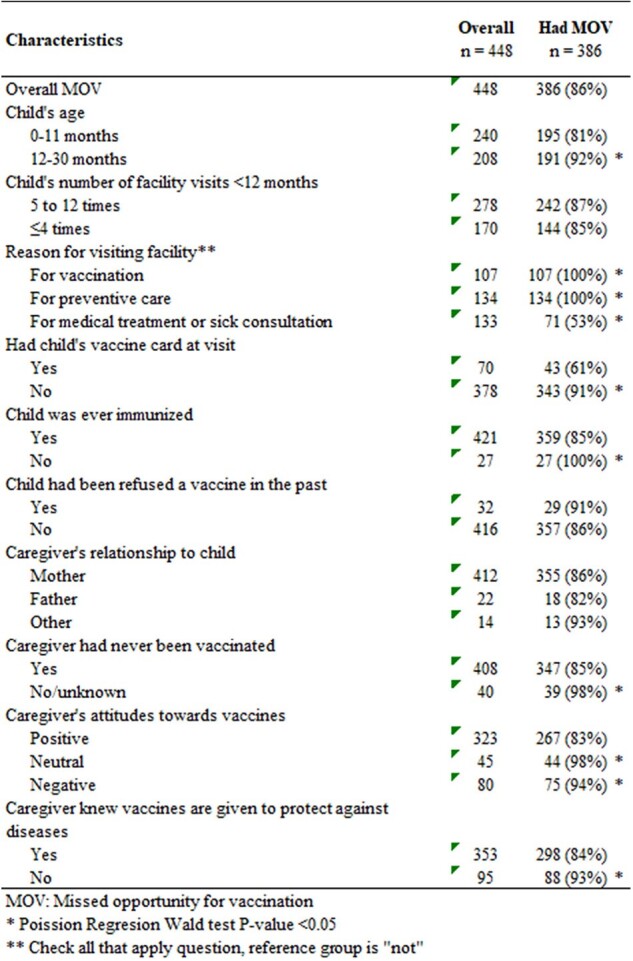
Characteristics associated with missed opportunities for vaccination among healthcare-seeking children in Kyrgzystan, 2023

**Results:**

Of 660 caregivers interviewed, 69% (448/650) met the inclusion criteria. MOV prevalence was 86% (386/448). In children aged 0-11 months, 81% (195/240) had MOV and in children 12–30-month-olds 92% (191/208) had MOV. Vaccine-specific MOV was lowest for hepatitis B vaccines (8%; 52/648) and highest for diphtheria- pertussis-tetanus vaccines (50%; 52/103) (Figure 1). Among eligible children (n=448), 62% had >4 annual clinic visits, 6% had never been immunized, and 84% were missing vaccine cards (Table 1). Healthcare providers did not check immunization status in 53% of children or ask to see vaccine cards in 79%. Among children who came specifically for vaccination, 53% (71/133) had a MOV. MOV did not differ by sex, district, or number of annual clinic visits. MOV was higher among children with never-vaccinated caregivers vs vaccinated caregivers (98% vs 85%, p=0.03) and caregivers with self-reported negative vaccine attitudes vs positive (94% vs 83%, p=0.01).

**Conclusion:**

Nearly nine out of ten children missing an opportunity to be immunized while seeking healthcare services. Additional training for healthcare workers, to check immunization status of all children at each visit can help reduce MOV. Strategies that address caregiver barriers to timely vaccinations can help increase immunization coverage.

**Disclosures:**

**All Authors**: No reported disclosures

